# Neighborhood opportunity, white matter, and cognition in a large pediatric neuroimaging study

**DOI:** 10.1038/s41398-026-04143-x

**Published:** 2026-06-05

**Authors:** Neslihan Yildiz-Ozhan, Benson S. Ku, Leo R. Zekelman, Ryan Zurrin, Fan Zhang, Lauren J. O’Donnell, Yogesh Rathi, Johanna Seitz-Holland, Suheyla Cetin-Karayumak

**Affiliations:** 1 Department of Psychiatry, Mass General Brigham, Harvard Medical School, Boston, USA; 2 Department of Psychiatry and Behavioral Sciences, Emory University School of Medicine, Atlanta, USA; 3 Department of Radiology, Mass General Brigham, Harvard Medical School, Boston, USA; 4 Speech and Hearing Bioscience and Technology, Harvard University, Cambridge, Massachusetts USA

**Keywords:** Neuroscience, Learning and memory

## Abstract

Neighborhood opportunities are key influences on child development, yet existing research has largely emphasized socioeconomic indicators as proxies for environmental context. However, it remains unclear which specific components of multidimensional neighborhood features are most strongly associated with tract-level white matter microstructure and cognition in children. Using data from 6141 children (ages 8–10 years) in the Adolescent Brain Cognitive Development (ABCD) Study and well-validated harmonized diffusion MRI, we examined multilevel associations between neighborhood opportunity, white matter microstructure, and cognitive performance. Neighborhood opportunity was measured using the Child Opportunity Index 2.0 (COI), which includes an overall score, three domain-level scores capturing Education, Social-Economic, and Health-Environment conditions, and 29 individual neighborhood indicators. Generalized additive models were used to characterize potentially non-linear associations while accounting for demographic and familial factors. Higher neighborhood opportunity was associated with greater fractional anisotropy (FA) in the Superior Longitudinal Fasciculus I (SLF-I), a fronto-parietal association tract implicated in higher-order cognitive functions. Higher neighborhood opportunity was also associated with better cognitive performance, with the strongest associations observed for Crystallized Cognition, reflecting accumulated knowledge and experience-based abilities. Mediation analyses indicated that SLF-I FA partially accounted for the association between neighborhood opportunity and cognitive performance. At the indicator level, access to high-quality education, higher neighborhood employment rates, and greater health insurance coverage emerged as the strongest correlates of neurocognitive outcomes. Together, these findings emphasize the value of multidimensional neighborhood measures and highlight specific, modifiable contextual factors that may represent targets for community-level interventions aimed at supporting neurocognitive development during childhood.

## Introduction

Neighborhood opportunities, such as access to quality education, socioeconomic stability, and healthcare resources, are believed to play a crucial role in shaping cognitive abilities, as well as physical and mental health outcomes across the lifespan [1–3]. These effects may be especially pronounced during childhood and adolescence, when the brain is highly sensitive to environmental inputs [4, 5]. Prior research shows that children from lower-opportunity neighborhoods tend to have poorer cognitive performance in language, processing speed, and executive function, and are at higher risk for psychiatric disorders [6–9]. Neuroimaging studies support these findings, revealing altered structural and functional brain development in key regions such as the frontal, parietal, temporal, and hippocampal regions in children from disadvantaged neighborhoods [10–13]. While these studies have linked neighborhood conditions to neurodevelopmental outcomes, neighborhood opportunity is a multidimensional construct that includes diverse factors across educational, economic, and environmental domains. However, it remains unclear which specific domains are most strongly associated with brain and cognitive development.

During childhood and adolescence, the brain undergoes significant structural and functional maturation, including myelination and the development of white matter tracts, which are essential for higher-order cognitive processes, such as memory, decision-making, and emotional regulation [14–16]. Among these, white matter association fiber tracts facilitate intra-hemispheric connectivity by linking key cortical areas—including the frontal, parietal, and temporal lobes—and are crucial for integrating sensory information, language, and executive functions [17–20]. Association fiber tracts mature later than projection and commissural pathways, with development extending into early adulthood, making them especially sensitive to environmental influences, including neighborhood-level exposures such as educational resources, socioeconomic conditions, and environmental quality [4, 21–23].

While existing research suggests that white matter development may be influenced by environmental factors, studies specifically examining the role of neighborhood opportunities in this process remain limited [1, 24, 25]. Furthermore, most prior research has often been constrained by small sample sizes and a narrow focus on socioeconomic factors, often neglecting broader domains such as educational access and environmental quality [1]. A recent study from the Adolescent Brain Cognitive Development (ABCD) Study found that early-life adversity was associated with lower fractional anisotropy (FA)—a diffusion MRI (dMRI)-based measure of white matter microstructure—and with reduced cognitive performance in language, inhibitory control, and mental arithmetic [25]. However, further work is needed to determine how specific, structural aspects of neighborhood opportunity relate to tract-specific white matter development and cognitive performance in large, population-based samples.

To address this gap, we examined the relationship between supportive neighborhood resources and white matter microstructure and cognitive performance in 6141 children (ages 8–10 years) using baseline data from the ABCD Study. We hypothesized that greater neighborhood opportunity would be associated with more developed white matter microstructure, reflected by higher FA values in association tracts, and with higher cognitive performance. Given prior work suggesting that neighborhood effects on cognition may operate through brain structure [19, 26, 27], we also tested whether FA serves as a mediating pathway linking neighborhood opportunity to cognitive performance, without inferring causality. Finally, we investigated which neighborhood opportunity domains—Education, Social-Economic, and Health-Environment—as well as their individual indicators, are linked to neurodevelopment.

## Methods

### Participants

**Fig. 1:**
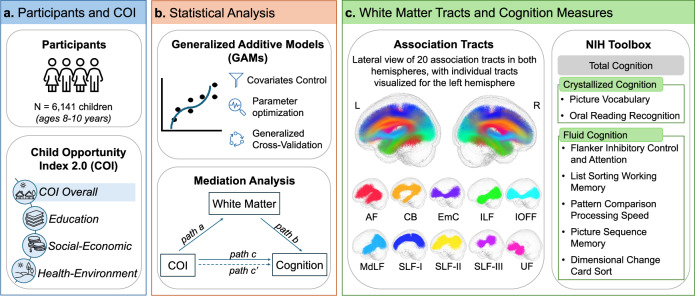
Study Overview. **a** ***Participants and COI:*** The analysis included 6141 children from the baseline data of the ABCD Study. Neighborhood opportunities were assessed using the Child Opportunity Index 2.0 (COI), a composite measure of children’s access to neighborhood resources. The COI captures multilevel features of the neighborhood environment through an Overall score, three domain-level scores—Education, Social-Economic, and Health-Environment—and 29 indicator-level scores that reflect specific neighborhood characteristics relevant to child development. **b** ***Statistical Analysis:*** Generalized Additive Models (GAMs) were applied to examine COI, white matter microstructure, and cognition associations. Mediation analyses were conducted to investigate the indirect effects of white matter microstructure on the COI-cognition associations. **c** ***White Matter and Cognitive Measures:*** White matter association fiber tracts were analyzed using harmonized, quality-controlled diffusion MRI data. Association tracts were visualized using the O’Donnell Research Group (ORG) tractography atlas in 3D Slicer [39–41, 75]. Cognitive performance was evaluated using the NIH Toolbox Cognitive Battery scores. L left, R right, AF arcuate fasciculus, CB cingulum bundle, EmC extreme capsule, ILF inferior longitudinal fasciculus, IOFF inferior occipital fasciculus, MdLF middle longitudinal fasciculus, SLF-I, II, III superior longitudinal fasciculus I, II, III, UF uncinate fasciculus.

This study used baseline data (*Release 5.1*) from the ABCD Study, a large-scale, longitudinal investigation of child health and neurodevelopment involving 11,868 children from 21 U.S. sites [28, 29]. For white matter analysis, harmonized dMRI data (*Release 3.0*) were used to minimize variability related to site and scanner differences [30]. After excluding individuals with missing data on neighborhood opportunity, harmonized dMRI data, cognitive scores, or required covariates (Table 1), the final sample included 6141 children (Supplementary Fig. 1). Participant characteristics are summarized in Table 1. An overview of the study design is shown in Fig. 1.

**Table 1:** Characteristics of the analyzed sample.

Characteristics *(N* = *6141)*	Means (SD) [Range]
**Age** *(in months)*	119.7 (7.5) [107 - 133]
**Child Opportunity Index 2.0 (COI) Scores**	
*Overall*	62.3 (29.8) [1 - 100]
*Education*	61.7 (29.2) [1 - 100]
*Social-Economic*	61.9 (30.1) [1 - 100]
*Health-Environmental*	59.5 (28.9) [1 - 100]
**NIH Toolbox Composite Scores**	
*Total Cognition Composite*	87.5 (8.8) [44 - 117]
*Fluid Cognition Composite*	92.8 (10.4) [44*–*131]
*Crystalized Cognition Composite*	87.3 (6.9) [58*–*115]
**Parental education** *(in years)*	16.1 (2.7) [3–22]
**Body Mass Index (BMI)**	18.6 (4.1) [5.6 - 54.9]
	**Participants, No. (%)**
**Sex**	
*Female*	2575 (41.9%)
*Male*	3566 (58.1%)
**Race**	
*White*	4827 (78.6%)
*Black*	892 (14.5%)
*Asian*	138 (2.3%)
*Other*^*a*^	284 (4.6%)
**Ethnicity**	
*Hispanic*	1114 (18.1%)
*Non-Hispanic*	5027 (81.9%)
**Household Income**	
*<25k*	807 (13.2%)
*25–49k*	879 (14.3%)
*50–74k*	878 (14.3%)
*75–99k*	874 (14.2%)
*100–199k*	1955 (31.8%)
*> 200k*	748 (12.2%)
**Pubertal Stage**	
*Prepuberty*	1708 (27.8%)
*Early puberty*	2444 (39.8%)
*Mid puberty*	1836 (29.9%)
*Late/Post puberty*	153 (2.5%)
**Sleep Hours**^b^	
*Less than 9* *h*	3097 (50.4%)
*More than 9* *h*	3044 (49.6%)
**Family History of Psychopathology**^c^	
*Yes*	2182 (35.5%)
*No*	3959 (64.5%)

*NIH* National Institute of Health.

^a^‘Other’ race category includes multiracial, indigenous, or unlisted racial groups, combined due to low participant counts.

^b^Based on American Academy of Sleep Medicine (AASM) recommendations for the respective age group, 9 h of sleep was used as the threshold for sufficient sleep [52].

^c^Family history of psychopathology indicates a history of schizophrenia, depression, mania, or hospitalization history due to mental health disorders in either biological parent.

### Neighborhood opportunity

The Child Opportunity Index 2.0 (COI) is a nationally-normed measure that captures children’s access to neighborhood resources using 29 indicators across 72,000 geographic areas in the U.S. [31–34].

In the ABCD Study, COI includes an Overall and three domain-level scores (Fig. 1a):

1. Education, including factors such as access to high-quality schools, school poverty rates, and teacher experience;
2. Social-Economic, reflecting aspects such as neighborhood economic resources, employment rates, and poverty levels;
3. Health-Environment, covering metrics such as health insurance coverage, walkability, and exposure to environmental toxins.

We used nationally normed COI Overall and domain scores (1–100), with higher scores indicating greater opportunity. Scores were assigned based on children’s primary residential addresses at baseline. For indicator-level analyses, each of the 29 indicators was z-scored within the ABCD sample. To ensure consistency in interpretation, indicators representing adverse conditions (e.g., poverty rates and toxin exposure) were reversed so that higher values consistently reflect greater opportunity. Full details of the 29 indicators are provided in Table 2 [32, 34].

**Table 2:** Child Opportunity Index 2.0 (COI) domains and indicators.

Early Childhood Education	Economic Opportunities	Healthy Environments
• Early childhood education centers	• Employment rate	• Access to healthy food
• High-quality early childhood education centers	• Commute duration	• Access to green space
• Early childhood education enrollment	**Economic and Social Resources**	• Walkability
**Elementary Education**	• Poverty rate*	• Housing vacancy rate
• Third grade reading proficiency	• Public assistance rate*	**Toxic Exposures**
• Third grade math proficiency	• Homeownership rate*	• Hazardous waste dump sites
**Secondary and Postsecondary Education**	• High-skill employment*	• Industrial pollutants in air, water, or soil
• High school graduation rate	• Median household income*	• Airborne microparticles
• Advanced Placement course enrollment	• Single-headed households	• Ozone concentration
• College enrollment in nearby institutions		• Extreme heat exposure
**Educational and Social Resources**		**Health Resources**
• School poverty		• Health insurance coverage
• Teacher experience		
• Adult educational attainment		

^*^These five indicators are combined into an economic resource index.

Some indicators are reversed when included in the index to ensure that higher values consistently represent greater opportunity.

### Association tracts microstructure using diffusion MRI data

Multi-shell dMRI data (b = 1000, 2000, 3000 s/mm^2^; 1.7 mm isotropic resolution) were collected using Siemens, GE, and Philips scanners across 21 ABCD sites. In our prior work [30], Philips data were excluded due to quality concerns, and harmonization was applied to Siemens and GE scans using our well-established method, the rotation-invariant spherical harmonics (RISH) method [35–37]. RISH harmonization reduces scanner-related signal variation by standardizing diffusion signal profiles, while preserving biologically meaningful inter-individual variability [35]. Compared to other harmonization approaches, RISH more effectively reduces scanner-induced bias without distorting underlying white matter architecture [38]. By improving cross-scanner comparability, this method enhances the reliability and validity of dMRI analyses in multi-site studies.

Following harmonization, whole-brain multi-fiber tractography was conducted to map white matter connections [30, 39, 40]. For each participant, 73 anatomically distinct white matter tracts were identified through white matter parcellation, enabling quantification of their microstructural properties [30, 41, 42]. The details are provided in our previous study [30]. For the present study, we focused on Fractional Anisotropy (FA) values from 20 long-range intra-hemispheric association fiber tracts (Fig. 1c) as proxies for white matter microstructure, neural pathway organization, and myelination to test our hypothesis [43].

### Neurocognitive assessment

Cognitive performance was assessed using the NIH Toolbox Cognitive Battery, which includes three composite scores and seven subtests (Fig. 1c) [44–46]. The Total Cognition Composite provides a comprehensive measure of general cognitive ability. The Crystallized Cognition Composite and its subtests, Picture Vocabulary and Oral Reading Recognition, evaluate knowledge-based skills acquired through learning and experience. The Fluid Cognition Composite and its subtests assess working memory (List Sorting Working Memory), processing speed (Pattern Comparison Processing Speed), inhibitory control and attention (Flanker Inhibitory Control and Attention), cognitive flexibility (Dimensional Change Card Sort), and episodic memory (Picture Sequence Memory). For this study, uncorrected standard scores were used, with all statistical analyses controlling for age and sex [47].

### Statistical analysis

We analyzed associations between neighborhood opportunity, white matter microstructure in association tracts, and cognitive performance using GAMs (Fig. 1b). To facilitate comparison, COI, cognition, and FA values were standardized into z-scores. GAMs [48, 49] were used to assess the effect of each COI score (*Overall*, domain-level, and indicator-level) on mean FA in each association tract and cognitive performance (NIH Toolbox). GAMs also examined the association between FA and cognition, adjusting for COI. Additionally, a mediation analysis was tested to determine whether FA mediates the relationship between neighborhood opportunity and cognition. Further details on the statistical methods are provided in the following subsections:

#### Covariate control

All analyses were adjusted for potential confounders, including age, sex, body mass index (BMI), sleep duration, pubertal stage, family psychopathology history, household income, parental education, and Family ID (to account for sibling status) [12, 15, 50–53]. Race and ethnicity were included in all models due to their strong associations with neighborhood opportunity rather than any presumed biological impact on neurodevelopment [31]. Site ID (21 study sites) was included as a covariate for cognitive outcomes to account for site-related variability, site or scanner differences in dMRI data were already addressed through harmonization [35]. For white matter analyses, head motion was additionally controlled to reduce motion-related artifacts that could affect FA measurements [54]. Multicollinearity was assessed using the Variance Inflation Factors (VIF <2) and pairwise correlation matrices to ensure covariates did not introduce redundancy/bias (Supplementary Fig. 2).

#### Modeling approach

GAMs were implemented in R (using the ‘mgcv’ package, version 4.3.1) [48]. GAMs flexibly model nonlinear relationships between predictors and outcomes using smooth functions.[49] Compared to traditional linear models [55], GAMs better capture complex data patterns without imposing strict linearity—an important consideration given non-linear FA trajectories observed in dMRI studies [23, 36]. Smooth terms were selected using the Akaike Information Criterion (AIC), with lower AIC values favoring models that achieve an optimal balance between goodness of fit and parsimony [49]. To further optimize smoothness levels and prevent overfitting, Generalized Cross-Validation (GCV) was applied to select smoothing parameters that minimize prediction error while penalizing unnecessary model complexity. Together, these complementary criteria ensure adequate model fit while maintaining interpretability of the estimated non-linear effects. The degree of non-linearity was quantified using effective degrees of freedom (EDF), with values near one indicating approximately linear associations and higher values reflecting increasing non-linear structure [48]. To control for Type I error, False Discovery Rate (FDR) correction (*p* <0.05) was applied to each GAM analysis [56].

Model performance was evaluated using adjusted R² and Deviance Explained, which quantified the proportion of variability explained and predictive ability. F-statistics quantified the contribution of COI scores. Partial effects with 95% confidence intervals (CI) were visualized using smooth curves. We also extracted the mean function—the fitted values of the outcome across the range of the predictor—and the mean slope, which represents the first derivative of the smooth term and captures the average rate of change in the outcome per unit change in the predictor across the model. Together, these estimates indicate the directionality and strength of the associations: positive slopes suggest that outcome values increase with higher opportunity scores, while negative slopes indicate inverse relationships. To directly address concerns regarding model fit and potential overfitting, we conducted a comprehensive set of model diagnostics (Supplementary Fig. 3). These included inspection of residual distributions and histograms to assess normality, evaluations of homoscedasticity to confirm stability of residual variance across fitted values, and examination of spline smoothness to ensure that non-linear terms were appropriately constrained and not driven by excessive model flexibility. Together, these diagnostics support the adequacy and robustness of the fitted GAMs.

#### Sensitivity analyses

To evaluate the robustness of the primary findings and address potential concerns regarding model flexibility, all key associations were additionally examined using linear mixed-effects models as sensitivity analyses. These models were implemented in R using the ‘lme4’ package [55] and included fixed effects for neighborhood opportunity and the same covariates used in the primary GAM analyses, along with random intercepts for family and site to account for sibling-relatedness and site-level clustering.

#### Mediation analyses

Mediation analysis examined whether white matter microstructure (FA in association tracts) statistically explained the associations between neighborhood opportunity and cognitive performance. Analyses were conducted using the ‘mediation‘ package in R, with indirect effects estimated via bootstrapping and 95% confidence intervals [57, 58]. The indirect effect (i.e., average causal mediation effect) was estimated using Monte Carlo simulations, which account for distribution differences between the mediator and outcome. Consistent with the cross-sectional design of the study, mediation analyses were implemented as a statistical decomposition of associations rather than as a test of causal mechanisms. Mediation proportions quantified the extent to which tract-level FA accounted for the observed COI-cognition relationships.

## Results

### Participant characteristics

After excluding individuals with missing data on neighborhood opportunity, harmonized dMRI, cognitive scores, or covariates (Table 1), the final sample included 6141 children (Supplementary Fig. 1), including 3566 boys (58.1%) with a mean age of 10.0 years (SD = 0.6). Participant characteristics are presented in Table 1, with corresponding ABCD Study variable names listed in Supplementary Table 1.

### Neighborhood opportunity and white matter microstructure

**Fig. 2:**
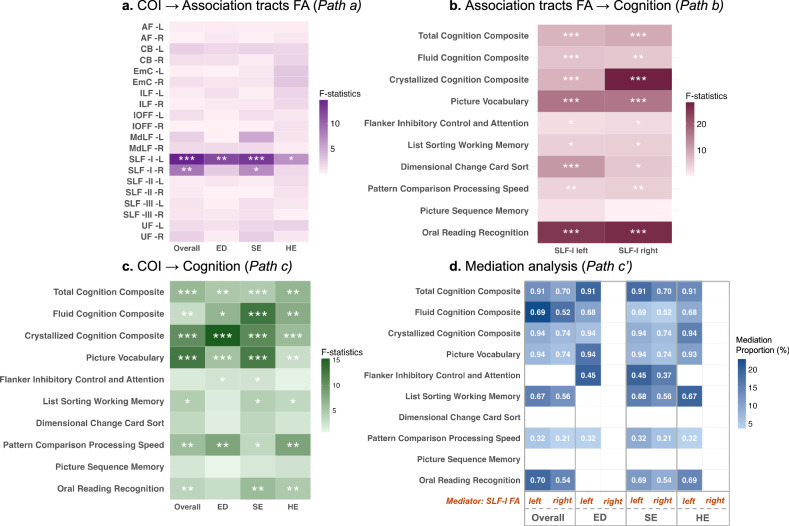
COI Overall and Domain-level Analysis. The heatmaps in *Path* ***a***, ***b***, *and* ***c*** represent independent GAM models for each Child Opportunity Index 2.0 (COI) score (*Overall*; ED: Education; SE: Social-Economic; HE: Health-Environment), which inform the mediation analysis (*Path c*′). Each column in (**a, b, and c**) represents a distinct GAMs result where the corresponding COI domain (x-axis) is the predictor. **F-statistics** indicate the strength of the association, with **significance levels** denoted as *p* <0.001 ‘***,’ *p* <0.01 ‘**,’ and *p* <0.05 ‘*,’ based on FDR-corrected *p*-values. **d** Mediation analysis was conducted for the positive associations identified in the GAMs, with each column representing a separate analysis of the associations between COI domains and cognition mediated by SLF-I FA (left or right). The heatmap displays the **indirect effects** of SLF-I FA in mediating the relationship between COI domains and cognitive outcomes, with darker shades indicating higher **mediation proportions**. Empty cells in the heatmap indicate where no prior associations were found in the GAMs model. FA: Fractional Anisotropy, -L left, -R right, AF arcuate fasciculus, CB cingulum bundle, EmC extreme capsule, ILF inferior longitudinal fasciculus, IOFF inferior occipital fasciculus, MdLF middle longitudinal fasciculus, SLF-I, II, III superior longitudinal fasciculus I, II, III, UF uncinate fasciculus.

We examined 20 association fiber tracts (10 per hemisphere) to evaluate their relationship with neighborhood opportunity using GAMs (Fig. 1c). Each COI score—Overall and domain-level—was modeled separately as a predictor of FA in its own GAM, with FDR correction applied across tracts (Supplementary Table 2). F-statistics and significance levels are visualized in the heatmap (Fig. 2a). Higher COI scores were significantly associated with greater FA only in the Superior Longitudinal Fasciculus-I (SLF-I) across all COI domains, suggesting that greater neighborhood opportunities support white matter development in this tract (Table 3a).

**Table 3:** Results of Generalized Additive Models (GAMs).

3a. COI → Association tracts (*Path a)*										
	COI → SLF – I FA (left)					COI → SLF – I FA (right)				
	EDF	R² (adj)/ Dev.Exp.	F	Mean Func./ Mean Slope	FDR corrected *p*-value	EDF	R² (adj)/ Dev.Exp.	F	Mean Func./ Mean Slope	FDR corrected *p*-value
COI Overall	1.00	0.09/9.6	14.63	0.29/0.07	**<0.001**	1.00	0.11/11.3	8.65	0.28/0.06	**0.006**
Education	1.02	0.09/9.6	11.95	0.31/0.06	**0.001**	2.63	0.11/11.0	2.57	0.30/0.04	0.08
Social-Economic	1.00	0.09/9.6	13.16	0.31/0.06	**<0.001**	1.00	0.11/11.3	7.09	0.30/0.05	**0.01**
Health-Environment	1.00	0.09/9.8	6.66	0.35/0.04	**0.02**	1.00	0.11/11.2	1.64	0.32/0.04	0.26
**3b. SLF – I FA → Cognition** ***(Path b)***										
	**SLF – I FA (left) → Cognition**					**SLF – I FA (right) → Cognition**				
Total Cognition Composite	4.39	0.33/33.9	7.96	0.49/0.09	**<0.001**	2.73	0.33/33.5	9.40	0.50/0.09	**<0.001**
Fluid Cognition Composite	4.46	0.22/22.3	5.90	0.32/0.07	**<0.001**	2.91	0.21/22.2	5.36	0.35/0.07	**0.002**
Crystallized Cognition Composite	3.10	0.32/32.5	7.80	0.45/0.09	**<0.001**	1.00	0.32/32.4	28.17	0.45/0.09	**<0.001**
Picture Vocabulary	1.00	0.30/30.2	16.50	0.46/0.10	**<0.001**	1.00	0.30/30.2	16.33	0.46/0.10	**<0.001**
Flanker Inhibitory Control and Attention	4.58	0.09/10.2	2.62	0.17/0.05	**0.02**	3.70	0.09/10.2	3.08	0.20/0.06	**0.02**
List Sorting Working Memory	2.55	0.14/14.2	4.33	0.44/0.05	**0.01**	1.76	0.14/14.2	4.55	0.45/0.05	**0.03**
Dimensional Change Card Sort	1.71	0.13/13.2	11.66	0.17/0.03	**<0.001**	2.54	0.12/13.1	5.49	0.19/0.03	**0.02**
Pattern Comparison Processing Speed	5.69	0.10/11.0	13.98	0.19/0.05	**0.001**	2.33	0.10/10.8	4.52	0.24/0.05	**0.009**
Picture Sequence Memory	6.95	0.08/9.2	2.03	0.13/0.03	0.09	5.07	0.08/8.9	0.13	0.11/0.02	0.85
Oral Reading Recognition	1.00	0.21/21.6	25.13	0.32/0.06	**<0.001**	2.91	0.21/21.4	25.87	0.32/0.06	**<0.001**

3a presents the associations between COI domains and SLF-I Fractional Anisotropy (FA) in the left and right hemispheres. 3b examines the relationship between left and right SLF-I FA and cognitive performance across NIH Toolbox scores while accounting for the effect of COI Overall, highlighting the independent contribution of SLF-I FA to cognitive outcomes. These GAM results inform the mediation analysis, where Path a represents the effect of COI on SLF-I FA, and Path b represents the relationship between SLF-I FA and cognitive performance.

The R² (adj) and Dev. Exp. values represent the total model performance, while the F-statistics reflect the specific effects of neighborhood opportunity. Mean Function, average fitted value; Mean Slope, average first derivative (direction of effect). Significant results represented in bold.

*FA* fractional anisotropy, *EDF* effective degrees of freedom, *R² (adj)* R² adjusted, *Dev. Exp*. deviance explained (%), *Mean Func*. mean function, *FDR* false discovery rate.

The association between *COI Overall* and SLF-I FA was stronger in the left hemisphere (R² = 0.09, F = 14.63, FDR-corrected *p* <0.001) compared to the right hemisphere (R² = 0.11, F = 8.65, FDR-corrected *p* = 0.006). The total GAM model explained 9% of the variance in left SLF-I and 11% in the right. However, the specific contribution of *COI Overall* was greater for the left SLF-I FA, as indicated by a higher F-statistic. Partial effects and confidence intervals of these associations are illustrated in Supplementary Fig. 4.

The left SLF-I FA showed significant positive associations across all COI domains—Education (R² = 0.09, F = 11.95, FDR-corrected *p* = 0.001), Social-Economic (R² = 0.09, F = 13.16, FDR-corrected *p* <0.001), and Health-Environment (R² = 0.09, F = 6.66, FDR-corrected *p* = 0.02)—whereas the right SLF-I exhibited a significant association only with the Social-Economic domain (R² = 0.11, F = 7.09, FDR-corrected *p* = 0.01), as shown in Fig. 2a. These findings indicate a domain-level influence of neighborhood opportunity on the SLF-I tract.

### Association between SLF-I tract and neurocognitive performance

After identifying positive associations between COI scores and SLF-I FA in both hemispheres, a second set of GAMs analysis was conducted to assess the relationship between SLF-I FA and cognitive performance (Table 3b), controlling for COI score and covariates.

Left and right SLF-I FA were each positively associated with higher Total Cognition and all cognitive subtests, except for Picture Sequence Memory (Fig. 2b). The strongest relationship was observed between right SLF-I and Crystallized Cognition, with the highest F-statistics (R² = 0.32, F = 28.17, FDR-corrected *p* <0.001). These results indicate that higher SLF-I FA is associated with superior cognitive performance.

### Neighborhood opportunity and neurocognitive performance

A third set of GAMs analyses examined the total effect of neighborhood opportunity on neurocognitive performance, modeling each COI domain as an individual predictor. FDR correction was applied across NIH Toolbox scores. The analysis revealed strong associations between higher neighborhood opportunity and greater neurocognitive performance, particularly in language, processing speed, and executive function. F-statistics and significance levels are visualized in Fig. 2c, with detailed results provided in Supplementary Table 3. Partial effect graphs illustrated these associations in Supplementary Fig.4. *COI Overall* was significantly associated with Total Cognition (R² = 0.32, F = 6.40, FDR-corrected *p* <0.001), as well as with several cognitive subtests (Path c, Table 4). The strongest relationship was observed with the Picture Vocabulary subtest, which demonstrated the highest F-statistic (F = 12.59). These findings suggest that greater neighborhood opportunity support improved cognitive performance.

**Table 4:** Results for neighborhood opportunity association with cognition and mediation analysis.

	COI Overall → Cognition Path c					Mediation Analysis Path c’			
	EDF	R² (adj)/ Dev.Exp.	F	Mean Func./ Mean Slope	FDR corrected *p*-value	(M: SLF – I Left)		(M: SLF – I Right)	
						Indirect Effect (95% CI)	Mediation proportion	Indirect Effect (95% CI)	Mediation proportion
Total Cognition Composite	4.4	0.32/33.0	6.40	0.54/0.09	**<0.001**	0.91 (0.87 – 0.96)	13.0%	0.70 (0.66 – 0.74)	9.9%
Fluid Cognition Composite	4.9	0.21/21.3	3.01	0.38/0.07	**0.004**	0.69 (0.65 – 0.72)	22.9%	0.52 (0.49 – 0.55)	17.3%
Crystallized Cognition Composite	2.9	0.32/32.0	10.39	0.48/0.10	**<0.001**	0.94 (0.90 – 0.99)	9.1%	0.74 (0.70 – 0.77)	7.1%
Picture Vocabulary	2.7	0.30/30.0	12.59	0.48/0.11	**<0.001**	0.94 (0.90 – 0.98)	7.5%	0.74 (0.71 – 0.78)	5.9%
Flanker Inhibitory Control and Attention	4.4	0.09/9.8	1.76	0.20/0.06	0.12	–	–	–	-
List Sorting Working Memory	1.4	0.13/13.9	4.45	0.46/0.05	**0.02**	0.68 (0.64 – 0.71)	15.2%	0.56 (0.53 – 0.58)	12.6%
Dimensional Change Card Sort	1.3	0.12/12.7	3.36	0.21/0.04	0.09	–	–	–	-
Pattern Comparison Processing Speed	1.6	0.10/10.1	5.92	0.26/0.05	**0.005**	0.32 (0.30 – 0.35)	5.5%	0.22 (0.19 – 0.24)	3.6%
Picture Sequence Memory	1	0.08/8.7	2.11	0.13/0.03	0.16	–	–	–	-
Oral Reading Recognition	4.2	0.21/21.3	3.71	0.34/0.07	**0.004**	0.70 (0.66 – 0.73)	18.7%	0.54 (0.51 – 0.57)	14.5%

*Path c* represents an independent Generalized Additive Model (GAM) assessing the direct effect of COI Overall on cognitive outcomes, which informs the mediation analysis (Path c′), presenting the indirect effects of SLF-I FA (left and right hemispheres) on cognitive outcomes, along with 95% confidence intervals (CI) and mediation proportions.

*FDR* false discovery rate, *EDF* effective degrees of freedom, *M* Mediator, *Dex. Exp* deviance explained, *R² (adj)* R² adjusted, *Mean Func*. mean function, *FA* fractional anisotropy, *CI* 95% confidence interval. Significant results are represented in bold. The R² (adj) and Dev. Exp. values represent the total performance of the models, while the F-statistics reflect the specific effects of neighborhood opportunity. Mean Function, average fitted value; Mean Slope, average first derivative (direction of effect). Significant results represented in bold.

The Education domain exhibited the strongest association with Crystallized Cognition (R^2^ = 0.31, F = 15.26, FDR-corrected *p* <0.001). Similarly, the Health-Environment domain was strongly associated with Crystallized Cognition (R^2^ = 0.31, F = 6.22, FDR-corrected *p* <0.001). The Social-Economic domain exhibited the highest association with Fluid Cognition (R^2^ = 0.31, F = 12.83, FDR-corrected *p* <0.001). Among cognition subtests, Picture Vocabulary consistently showed the strongest associations across COI domains (Fig. 2c).

### Sensitivity analyses

To assess the robustness of the primary findings, all associations were additionally examined using linear mixed-effects models. Results were consistent with those obtained from the GAM framework, with the SLF-I remaining the only association tract showing a statistically significant relationship with neighborhood opportunity (Supplementary Table 4). Specifically, higher *COI Overall* was associated with greater FA in the SLF-I left (R² = 0.09, β = 0.05, FDR-corrected *p* <0.01), and the strongest cognitive association was observed for Crystallized Cognition (R² = 0.31, β = 0.08, FDR-corrected *p* <0.001). The direction and relative magnitude of the SLF-I and cognition effect estimates were consistent across modeling approaches. Importantly, statistical inference was unchanged after accounting for family-relatedness and site-level clustering via random intercepts. These findings support the robustness of the primary results and indicate that they are not driven by the flexibility of the GAM framework. Notably, the SLF-I remained the only association tract significantly related to neighborhood opportunity in linear mixed-effects models, indicating that tract specificity was preserved under alternative modeling assumptions.

### Mediation analyses

Mediation analyses assessed whether SLF-I FA mediated the positive association between neighborhood opportunities and cognitive outcomes (Fig. 1b). Significant indirect effects were observed for SLF-I (Path c′, Table 4) across all COI domains, with mediation proportions ranging from 3.6–22.9% (Supplementary Table 3). Figure 2d presents a heatmap summarizing all mediation results.

The left SLF-I FA showed a significant indirect effect on Total Cognition (0.91, 95% CI: 0.87–0.96) with a 13% mediation proportion, indicating that 13% of the total effect of *COI Overall* on cognition was mediated through left SLF-I FA. These findings underscore the partial mediating role of SLF-I FA in the relationship between neighborhood opportunities and cognitive outcomes.

### Neighborhood opportunity indicator-level analyses

To identify which specific neighborhood indicators were most strongly associated with neurocognitive development, we examined associations between the 29 COI indicators and SLF-I FA, as well as cognitive outcomes that demonstrated significant associations in the domain-level analyses. GAMs were used for each COI indicator as a predictor, adjusting for covariates.

Within the indicator-level analysis, adult educational attainment (R^2^ = 0.32, F = 33.48, FDR-corrected *p* <0.001), public assistance rate (reversed; R^2^ = 0.32, F = 31.30, FDR-corrected *p* <0.001), and employment rate (R^2^ = 0.32, F = 30.69, FDR-corrected *p* <0.001) showed the top 3 strongest associations with Total Cognition and highlight the indicators of Social-Economic and Educational domain association with cognitive performance. In the Health-Environment domain indicators, health insurance coverage (R^2^ = 0.32, F = 9.79, FDR-corrected *p* <0.001) emerged as the most robust correlate.

**Fig. 3:**
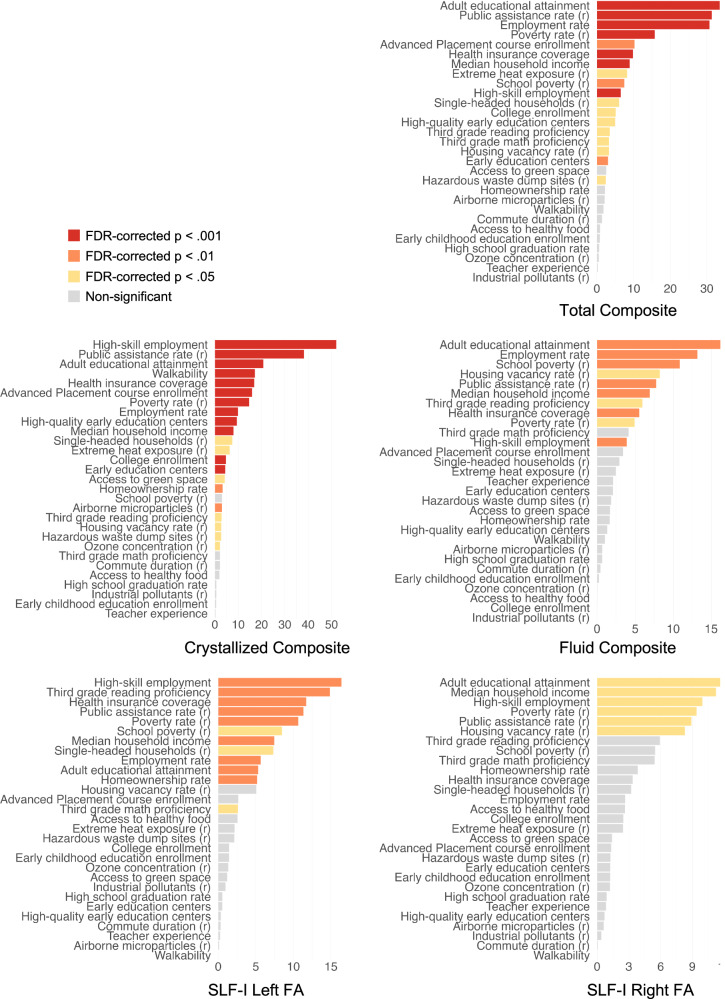
COI Indicator-level Analysis. Associations between 29 Child Opportunity Index 2.0 (COI) indicators and both cognitive composite scores and SLF-I fractional anisotropy (FA) values. The bar plot displays F-statistics derived from generalized additive models (GAMs) for each COI indicator. Indicators marked with “(r)” were reversed so that higher values consistently reflect greater neighborhood opportunity.

Consistent with our neighborhood domain analyses, these indicator-level findings reinforce the observation that Crystallized Cognition—reflecting accumulated language and reading skills—is more strongly associated with neighborhood opportunity indicators than Fluid Cognition (Fig. 3).

For SLF-I, multiple neighborhood indicators were significantly and positively associated with higher FA values, with stronger associations observed in the left hemisphere, consistent with domain-level analysis. The strongest associations with left SLF-I FA were observed for indicators of different neighborhood domains, including high-skill employment rates (R^2^ = 0.06, F = 16.40, FDR-corrected *p* <0.01), third-grade reading proficiency (R^2^ = 0.06, F = 14.85, FDR-corrected *p* <0.01), and health insurance coverage (R^2^ = 0.06, F = 11.71, FDR-corrected *p* <0.01), showing that white matter microstructure is also associated with multiple dimensions of neighborhood opportunity. Figure 3 summarizes the indicator-level associations with composite cognitive scores and SLF-I FA, while results for cognitive subtests are provided in Supplementary Fig. 5.

## Discussion

To our knowledge, this study is among the most comprehensive to examine the associations between neighborhood opportunity—measured at the Overall, domain, and indicator levels using the COI—, white matter microstructure in association tracts, and cognitive performance in children. Our findings demonstrate that lower neighborhood opportunity is associated with reduced SLF-I FA and poorer cognitive performance. SLF-I FA partially mediates this relationship, indicating a potential neurobiological pathway linking educational, socioeconomic, and environmental resources with neurocognitive abilities. Furthermore, our indicator-level analysis reveals that specific neighborhood features—such as high-quality early education, employment rates, adult educational attainment, and healthcare access—are particularly relevant to neurodevelopmental outcomes, offering more precise targets for policy and intervention.

Previous studies have linked neighborhood disadvantages to alterations in cortical regions, including the superior parietal cortex, rostral middle frontal cortex, and precuneus, all of which are connected by SLF-I [8, 12, 17, 24, 59]. While a few dMRI studies have also reported lower FA in association tracts such as the arcuate and uncinate fasciculi and the SLF, these findings have primarily focused on global socioeconomic disadvantages [24, 60] or cumulative adversity [25]. In contrast, our study advances this literature by examining a structured, multidimensional neighborhood framework, allowing for more precise insights into the environmental correlates of neurodevelopment.

The SLF-I emerged as the primary tract most consistently associated with neighborhood opportunity in our analysis. This may reflect methodological strengths of the study, including a larger sample size, the use of harmonized dMRI data to reduce scanner-related variability, flexible modeling approaches that capture non-linear relationships, and comprehensive covariate adjustment, which together enhance sensitivity to subtle, tract-specific associations. The absence of significant associations in other tracts does not preclude their involvement, particularly at different developmental stages or under alternative environmental exposures. Differences in age ranges and neighborhood metrics used in other studies may also contribute to variability in reported findings [9, 25, 50].

The SLF-I tract, a dorsal component of the Superior Longitudinal Fasciculus, supports higher-order cognitive functions including language, working memory, and voluntary orienting of attention [17, 61]. Our results show that higher COI scores are associated with greater SLF-I FA, suggesting that children in higher-opportunity environments may exhibit more developed white matter organization in this tract. Notably, the left SLF-I showed stronger associations with neighborhood opportunity, consistent with the left hemisphere’s role in language, attention, and executive function [62, 63].

Higher COI scores were also positively associated with improved cognitive performance, especially in Crystallized Cognitive functions. These findings are consistent with the notion that access to quality educational, social, and health resources contributes to stronger language and accumulated knowledge-based cognitive abilities [1, 7, 64, 65]. In contrast, children from lower-opportunity neighborhoods are often exposed to chronic stress, violence, and environmental hazards that may impair neurodevelopment [66, 67]. Mediation analyses further suggest that SLF-I FA may partially link neighborhood opportunities and cognitive outcomes, highlighting a potential structural correlate of environmental variation in cognition.

Taken together, the involvement of the SLF-I may reflect an alignment between its functional role and developmental timing. The SLF-I connects dorsal fronto-parietal regions implicated in language, executive control, and attentional regulation—cognitive domains that overlap with those supported by educational access and cognitively enriching environments. Its prolonged maturation across late childhood and adolescence may increase its sensitivity to sustained environmental influences during this developmental window. In contrast, other association tracts may be more strongly shaped by earlier developmental processes or different experiential contexts, which may contribute to their weaker or less consistent associations with neighborhood opportunity in the present age range.

Altered SLF microstructure has also been reported in psychiatric and neurodevelopmental conditions such as schizophrenia, bipolar disorder, depression, anxiety, autism spectrum disorder, and attention deficit hyperactivity disorder [36, 68–71]. This is notable given the developmental timing of SLF maturation and its relevance to a range of cognitive and emotional functions [5, 21–23]. Although our sample comprised typically developing children, the observed associations, together with the prolonged developmental trajectory of the SLF, suggest that this tract may be particularly sensitive to environmental conditions. While causality cannot be inferred, SLF-I microstructure may serve as a structural marker of experience-dependent environmental variation with potential long-term relevance for cognitive and mental health outcomes [1, 25].

Domain-level analyses revealed distinct patterns of associations between specific COI domains and cognitive outcomes. The Social-Economic domain showed the strongest associations, significantly shaping both Crystallized and Fluid Cognition, underscoring the importance of neighborhood socioeconomic stability in cognitive development [1]. The Education domain was particularly linked to language and executive function, while the Health-Environment domain showed stronger associations with processing speed, emphasizing the role of environmental quality in cognitive efficiency [7, 64, 72]. Notably, Picture Vocabulary consistently exhibited the strongest associations across COI domains, reinforcing the critical role of neighborhood opportunities in fostering language development [9, 73].

Indicator-level analyses further demonstrated that neighborhood resources are not uniformly related to neurocognitive development. Specific neighborhood characteristics—particularly within the Social-Economic and Education domains—showed the strongest and most consistent associations with both SLF-I FA and cognitive outcomes. Notably, indicators such as access to high-quality early education, adult educational attainment, employment rates, and health insurance coverage were among the most robust correlates of neurocognitive outcomes, particularly in Crystallized Cognition. Together, these indicators capture key aspects of the cognitive, social, and material contexts in which children develop, providing a more granular understanding of how neighborhood-level conditions relate to variability in brain structure and cognitive performance beyond global socioeconomic measures.

More specifically, access to high-quality education and cognitively enriching environments may be associated with greater exposure to language, learning activities, and structured cognitive demands, which are closely linked to the development of executive and language-related functions. These experiences are relevant for fronto-parietal white matter organization, including pathways such as the SLF-I that support these cognitive domains [12, 72]. Employment stability and broader socioeconomic resources may shape children’s developmental context by influencing household stress levels, daily routines, and access to supportive resources, with lower exposure to psychosocial stressors being associated with more favorable neurocognitive outcomes [66, 67, 74]. In addition, access to health insurance and healthcare services may relate to earlier recognition and management of medical, developmental, or learning-related concerns, which can influence cognitive functioning and brain development during childhood [66, 67, 74]. Considered together, these indicators appear to operate as interconnected aspects of children’s lived environments, rather than acting in isolation, emphasizing the importance of evaluating neighborhood conditions as a broader constellation of multidimensional factors when examining differences in neurodevelopmental risk and resilience.

Together, these findings identify modifiable, community-level features—such as early education access, adult educational attainment, employment stability, and healthcare infrastructure—as promising targets for public health and education policy. These indicators align with existing policy domains that include early childhood initiatives, adult education programs, economic development efforts, and expanded healthcare access. The inclusion of both child- and adult-oriented indicators suggests that cross-generational investments may foster enriched developmental contexts that support cognitive outcomes [12]. By moving beyond global socioeconomic metrics to pinpoint specific, actionable factors, this study offers a more precise framework for guiding neighborhood-level interventions that promote child development and reduce health disparities.

Despite the strengths of this study, several limitations should be considered. First, the cross-sectional design limits causal interpretation by preventing conclusions about temporal ordering and directionality. While mediation analyses offer insight into potential linking mechanisms, longitudinal data are required to establish causal pathways and long-term effects. Additionally, only baseline dMRI data were harmonized and processed in this study [30]. Due to the computational complexity and significant resources required for large-scale harmonization, follow-up dMRI data have not yet been processed. However, these efforts are planned for future work to enable longitudinal analyses of white matter development over time. Second, although the ABCD Study includes participants from a wide range of neighborhood contexts, the analytic sample skewed toward higher socioeconomic status, with relatively few children exposed to extreme deprivation. While the subset of participants from lower-opportunity neighborhoods was sufficiently large to support robust statistical analyses (Supplementary Fig. 6), this distribution may limit the generalizability of the findings to populations experiencing the most severe structural disadvantage. Third, neighborhood opportunity was assessed using the Child Opportunity Index, which represents a static, cross-sectional measure and does not capture dynamic changes in neighborhood conditions or residential mobility over time. Consequently, the observed associations should be interpreted as reflecting proximal neighborhood context at baseline rather than cumulative or time-varying exposure. Future research incorporating longitudinal neighborhood data alongside individual- and family-level measures will be critical for better characterizing the dynamic, multilevel influences of environmental context on neurodevelopment. Lastly, although we adjusted for household income, parental education, and family psychiatric history, residual confounding and potential selection effects cannot be ruled out. It remains possible that children with higher cognitive abilities live in more enriched environments due to parental choices or mobility. Longitudinal designs are needed to clarify directionality and rule out reverse causation.

In conclusion, this study provides robust evidence linking neighborhood opportunity to white matter microstructure and cognitive performance in children. Across domains and indicators, we identify neighborhood factors associated with neurodevelopment, offering insight into where targeted, community-level interventions may be most impactful.

By identifying the SLF-I as a key tract linked to neighborhood conditions—and as a partial mediator of the relationship between neighborhood opportunity and cognition—this study highlights a structural neurodevelopmental correlate of environmental variation. Interventions focused on increasing access to early education, adult education, economic stability, and healthcare may support neurocognitive development and reduce disparities. Future longitudinal research will be essential to clarify these pathways and inform effective, equity-focused public health and education strategies.

## Supplementary information

Supplementary Information: Neighborhood Opportunity, White Matter, and Cognition in a Large Pediatric Neuroimaging Study

## Data Availability

Researchers interested in accessing the data can visit the National Institutes of Health ABCD study site (https://nda.nih.gov/abcd/). Further details on the harmonization method are available in our previous study (https://www.nature.com/articles/s41597-024-03058-w).

## Code availability

The data processing and statistical analysis code for this study can be found at https://github.com/neslihanyildizozhan/ABCD-COI.git. The multi-site diffusion MRI harmonization code is available at https://github.com/pnlbwh/dMRIharmonization.
