# Supplementary material for: Neighborhood opportunity, white matter, and cognition in a large pediatric neuroimaging study
Source: Transl Psychiatry. 2026 Jun 5;16:404. doi: 10.1038/s41398-026-04143-x (PMC13454431; doi:10.1038/s41398-026-04143-x)
Supplement: Supplementary file 1 [file 41398_2026_4143_MOESM1_ESM.docx]

## **Supplementary Information**

**Title: Neighborhood Opportunity, White Matter, and Cognition in a Large Pediatric Neuroimaging Study**

**Supplementary Table 1. ABCD Study Variables:** Table, Variable names, descriptions, and categories.

**Supplementary Table 2:** Generalized additive models result of Child Opportunity Index (COI) domains association with 20 association fiber tracts.

# **Supplementary Table 3**. GAMs result of the COI domains associated with cognition (path c) and mediation analysis (path c’).

**Supplementary Table 4.** Sensitivity analyses using linear mixed-effects models.

# **Supplementary Fig. 1.** The figure displays the number of subjects excluded due to missing values.

# **Supplementary Fig. 2.** Correlation Matrix and Variance Inflation Factors (VIF) for Included Variables to Assess Multicollinearity

# **Supplementary Fig. 3.** Diagnostic plots assessing the adequacy of the GAMs.

# **Supplementary Fig. 4:** These figures display the partial effect of COI Overall on both cognitive function tests and SLF-I tracts within GAMs. The smooth line indicates the direction of the association, with a 95% confidence interval.

**Supplementary Fig. 5.** Associations between 29 COI indicators and NIH Toolbox Cognitive scores

**Supplementary Fig. 6.** Scatter plot shows the distribution of Child Opportunity Index 2.0 (COI) Overall score across NIH Toolbox Total Cognition Composite.

**Supplementary References.**

**Supplementary Table 1. ABCD Study Variables.** Table, Variable names, descriptions, and categories.^1,2^

| **Variables** | **Table Name** | **Variable Name** | **Description** |
| --- | --- | --- | --- |
| ***Child Opportunity Index 2.0 (COI)*** | led_l_coi |  |  |
| COI Overall | led_l_coi | reshist_addr1_coi_r_coi_nat | nationally normed raw scores |
| COI Social-Economic | led_l_coi | reshist_addr1_coi_r_se_nat | nationally normed raw scores |
| COI Health-Environment | led_l_coi | reshist_addr1_coi_r_he_nat | nationally normed raw scores |
| COI Education | led_l_coi | reshist_addr1_coi_r_ed_nat | nationally normed raw scores |
| **Age (in months)** | abcd_y_lt | interview_age |  |
| ***Sex*** | abcd_p_demo | demo_sex_v2 |  |
| ***Race*** | abcd_p_demo | demo_race_a_p___ | Categorized as White, Black, Asian and Other |
| **Ethnicity** | abcd_p_demo | demo_ethn_v2 | Categorized as Hispanic and Non-hispanic |
| ***Parent Education (in years)*** ^a^ | abcd_p_demo | demo_prnt_ed_v2, demo_prtnr_ed_v2 | Parent education categories converted into years of education |
| **Household Income** | abcd_p_demo | demo_comb_income_v2 | Categorized as: <25k, 25-49k, 50-74k, 75-99k, 100-199k, >200k |
| ***Puberty Stages*** | ph_y_pds | pds_y_ss_female_category_2, pds_y_ss_male_cat_2 | Pubertal Developmental Scale Youth, based on sex at birth. Categorized as Prepuberty, Early puberty, Mid puberty, Late/ Post puberty |
| ***BMI*** | ph_y_anthro | anthroheightcalc, anthroweightcalc | BMI Calculation = weight (lb) / [height (in)]2 x 703 |
| ***Sleep*** | ph_p_sds | sleepdisturb1_p | Categorized as less than 9 hours, more than 9 hours |
| ***Family Psychopathology History*** | mh_p_fhx | fam_history_q7a_mania, fam_history_q7d_mania,fam_history_q6a_depression, fam_history_q6d_depression, fam_history_q12a_hospitalized, fam_history_q12d_hospitalized, fam_history_q8a_visions, fam_history_q8d_visions,  fam_history_q10d_nerves','fam_history_q10a_nerves | Categorized as a binary variable (1 = history of psychopathology in either biological parent). |
| **SITE ID** | abcd_y_lt | site_id |  |
| **FAMILY ID** | abcd_y_lt | family_id |  |
| **Head Motion** | mri_y_qc_motion | dmri_meanmotion |  |
| **NIH Toolbox** |  |  | Uncorrected standardized scores used |
| Total Cognition Composite | nc_y_nihtb | nihtbx_totalcomp_uncorrected |  |
| Crystallized Cognition Composite | nc_y_nihtb | nihtbx_cryst_uncorrected |  |
| Fluid Cognition Composite | nc_y_nihtb | nihtbx_fluidcomp_uncorrected |  |
| Picture Vocabulary | nc_y_nihtb | nihtbx_picvocab_uncorrected |  |
| Flanker Inhibitory Control and Attention | nc_y_nihtb | nihtbx_flanker_uncorrected |  |
| List Sorting Working Memory | nc_y_nihtb | nihtbx_list_uncorrected |  |
| Dimensional Change Card Sort | nc_y_nihtb | nihtbx_cardsort_uncorrected |  |
| Pattern Comparison Processing Speed | nc_y_nihtb | nihtbx_pattern_uncorrected |  |
| Picture Sequence Memory | nc_y_nihtb | nihtbx_picture_uncorrected |  |
| Oral Reading Recognition | nc_y_nihtb | nihtbx_reading_uncorrected |  |

^a^ **Parent education categories converted into years of education:**

**Categories**	**Education Level**	**Years of Education**

0	Never attended/Kindergarten	0 years

1	1st grade	1 year

2	2nd grade	2 years

3	3rd grade	3 years

4	4th grade	4 years

5	5th grade	5 years

6	6th grade	6 years

7	7th grade	7 years

8	8th grade	8 years

9	9th grade	9 years

10	10th grade	10 years

11	11th grade	11 years

12	12th grade	12 years

13	High school graduate	12 years

14	GED or equivalent diploma	12 years

15	Some college	14 years

16	Associate degree (Occupational)	14 years

17	Associate degree (Academic Program)	14 years

18	Bachelor’s degree	16 years

19	Master’s degree	18 years

20	Professional degree (e.g., MD)	20 years

21	Doctoral degree (e.g., PhD)	22 years

777	Refused to answer	N/A

**Supplementary Table 2.** Generalized Additive models (GAMs)[^3,4^](https://paperpile.com/c/6gKfd1/g5W7+ZfXj) result of the Child Opportunity Index (COI)[^5,6^](https://paperpile.com/c/6gKfd1/s6BB+6Eao) domains association with harmonized, quality-controlled association fiber tracts.^7^

***2a.*** *COI Overall -> Association tracts*

|  | **COI Overall** | | | | | | |
| --- | --- | --- | --- | --- | --- | --- | --- |
| *Association Tracts FA* | **EDF** | **R-sq. (adj)** | **Dev Exp** | **F** | **Mean Func. / Mean Slope** | **p-value** | **FDR corrected p-value** |
| AF -L | 1.00 | 0.16 | 16.4% | 0.35 | 0.15/0.02 | .55 | .60 |
| AF -R | 1.00 | 0.10 | 10.2% | 0.00 | 0.02/0.01 | .97 | .97 |
| CB -L | 2.22 | 0.14 | 14.6% | 2.30 | 0.14/-0.01 | .06 | .09 |
| CB -R | 1.83 | 0.13 | 13.3% | 1.23 | 0.11/0.00 | .29 | .35 |
| EmC -L | 1.00 | 0.14 | 14.8% | 0.15 | 0.08/0.01 | .70 | .74 |
| EmC -R | 1.00 | 0.14 | 14.4% | 1.12 | 0.1/-0.01 | .29 | .35 |
| ILF -L | 1.00 | 0.10 | 10.6% | 0.43 | 0.18/-0.01 | .51 | .57 |
| ILF -R | 1.00 | 0.10 | 10.2% | 0.18 | 0.09/0.01 | .67 | .71 |
| IOFF -L | 1.00 | 0.17 | 17.8% | 1.18 | 0.01/0.03 | .28 | .34 |
| IOFF -R | 1.00 | 0.17 | 17.3% | 0.03 | 0.01/0.01 | .86 | .87 |
| MdLF -L | 1.00 | 0.14 | 14.2% | 2.28 | -0.05/0.03 | .13 | .19 |
| MdLF -R | 1.85 | 0.15 | 15.4% | 1.49 | -0.02/0.03 | .26 | .33 |
| SLF -I -L | 1.00 | **0.09** | **9.6%** | **14.63** | **0.29/0.07** | **< 0.001** | **< 0.001** |
| SLF -I -R | 1.00 | **0.11** | **11.3%** | **8.65** | **0.28/0.06** | **.003** | **.006** |
| SLF -II -L | 1.31 | 0.10 | 9.9% | 0.41 | 0.23/0.02 | .74 | .76 |
| SLF -II -R | 3.08 | 0.14 | 14.9% | 0.47 | 0.12/0.02 | .66 | .71 |
| SLF -III -L | 1.00 | 0.10 | 10.1% | 0.91 | 0.14/0.00 | .34 | .40 |
| SLF -III -R | 1.00 | 0.11 | 11.2% | 0.83 | 0.10/0.02 | .36 | .42 |
| UF -L | 1.00 | 0.08 | 8.1% | 1.47 | 0.16/0.03 | .23 | .29 |
| UF -R | 1.00 | 0.09 | 9.5% | 2.23 | 0.08/0.03 | .14 | .19 |
| *Abbreviations:* FA:Fractional Anisotropy; EDF: Effective degrees of freedom-L: left; -R: right; AF: arcuate fasciculus; CB: cingulum bundle; EmC: extreme capsule; ILF: inferior longitudinal fasciculus; IOFF: inferior occipital fasciculus; MdLF: middle longitudinal fasciculus; SLF-I, II, III: superior longitudinal fasciculus I, II, III; UF: uncinate fasciculus | | | | | | | |

***2b.*** *COI Education -> Association tracts*

|  | **COI Education** | | | | | | |
| --- | --- | --- | --- | --- | --- | --- | --- |
| *Association Tracts FA* | **EDF** | **R-sq. (adj)** | **Dev Exp** | **F** | **Mean Func. / Mean Slope** | **p-value** | **FDR corrected p-value** |
| AF -L | 1.00 | 0.16 | 16.4% | 0.41 | 0.15/0.01 | .52 | .60 |
| AF -R | 2.78 | 0.10 | 10.4% | 0.61 | 0.03/0.01 | .56 | .62 |
| CB -L | 2.69 | 0.14 | 14.6% | 1.72 | 0.17/-0.03 | .14 | .20 |
| CB -R | 1.00 | 0.13 | 13.3% | 1.09 | 0.13/-0.01 | .30 | .39 |
| EmC -L | 1.00 | 0.14 | 14.8% | 0.03 | 0.10/0.00 | .87 | .90 |
| EmC -R | 1.55 | 0.14 | 14.4% | 0.83 | -0.01/-0.02 | .50 | .58 |
| ILF -L | 3.00 | 0.10 | 10.7% | 1.15 | 0.19/0.00 | .30 | .39 |
| ILF -R | 1.52 | 0.10 | 10.3% | 0.49 | 0.10/0.00 | .65 | .71 |
| IOFF -L | 1.00 | 0.17 | 17.8% | 0.14 | 0.03/0.01 | .71 | .77 |
| IOFF -R | 1.00 | 0.17 | 17.3% | 0.00 | 0.02/0.00 | .96 | .97 |
| MdLF -L | 1.00 | 0.14 | 14.2% | 0.00 | -0.01/0.00 | .97 | .97 |
| MdLF -R | 2.61 | 0.15 | 15.4% | 1.51 | -0.01/0.02 | .20 | .27 |
| SLF -I -L | 1.00 | **0.09** | **9.6%** | **11.95** | 0.31/0.06 | **< .001** | **.001** |
| SLF -I -R | 2.63 | 0.11 | 11.0% | 2.57 | 0.30/0.04 | .05 | .08 |
| SLF -II -L | 1.00 | 0.10 | 9.9% | 0.74 | 0.22/0.02 | .39 | .48 |
| SLF -II -R | 1.00 | 0.14 | 14.8% | 0.00 | 0.13/0.00 | .95 | .97 |
| SLF -III -L | 1.12 | 0.10 | 10.1% | 0.13 | 0.12/0.01 | .88 | .91 |
| SLF -III -R | 1.00 | 0.10 | 10.8% | 0.62 | 0.11/0.01 | .43 | .52 |
| UF -L | 1.00 | 0.08 | 8.6% | 1.02 | 0.15/0.02 | .31 | .40 |
| UF -R | 1.11 | 0.09 | 9.5% | 0.37 | 0.10/0.01 | .53 | .60 |
| *Abbreviations:* FA:Fractional Anisotropy; EDF: Effective degrees of freedom-L: left; -R: right; AF: arcuate fasciculus; CB: cingulum bundle; EmC: extreme capsule; ILF: inferior longitudinal fasciculus; IOFF: inferior occipital fasciculus; MdLF: middle longitudinal fasciculus; SLF-I, II, III: superior longitudinal fasciculus I, II, III; UF: uncinate fasciculus | | | | | | | |

***2c.*** *COI Social-Economic -> Association tracts*

|  | **COI Social-Economic** | | | | | | |
| --- | --- | --- | --- | --- | --- | --- | --- |
| *Association Tracts FA* | **EDF** | **R-sq. (adj)** | **Dev Exp** | **F** | **Mean Func. / Mean Slope** | **p-value** | **FDR corrected p-value** |
| AF -L | 1.00 | 0.16 | 16.4% | 0.29 | 0.15/0.02 | .59 | .64 |
| AF -R | 1.00 | 0.10 | 10.2% | 0.04 | 0.03/0.01 | .84 | .86 |
| CB -L | 2.70 | 0.14 | 14.5% | 2.00 | 0.13/-0.01 | .12 | .16 |
| CB -R | 1.49 | 0.13 | 13.2% | 0.39 | 0.11/0.02 | .71 | .75 |
| EmC -L | 1.00 | 0.14 | 14.9% | 0.68 | 0.08/-0.01 | .41 | .47 |
| EmC -R | 1.00 | 0.14 | 14.4% | 0.78 | -0.01/0.00 | .38 | .44 |
| ILF -L | 1.00 | 0.10 | 10.6% | 0.16 | 0.18/0.02 | .69 | .74 |
| ILF -R | 1.00 | 0.10 | 10.3% | 0.60 | 0.08/0.03 | .44 | .50 |
| IOFF -L | 1.75 | 0.17 | 17.9% | 1.26 | 0.02/0.03 | .27 | .33 |
| IOFF -R | 1.00 | 0.17 | 17.3% | 0.04 | 0.01/0.01 | .84 | .86 |
| MdLF -L | 1.00 | 0.14 | 14.3% | 5.00 | -0.05/0.04 | .03 | .05 |
| MdLF -R | 3.83 | 0.15 | 15.4% | 1.53 | -0.02/0.03 | .20 | .26 |
| SLF -I -L | 1.00 | **0.09** | **9.6%** | **13.16** | 0.31/0.06 | **< .001** | **< .001** |
| SLF -I -R | 1.00 | **0.11** | **11.3%** | **7.09** | 0.30/0.05 | **.008** | **.01** |
| SLF -II -L | 1.35 | 0.09 | 9.9% | 0.35 | 0.24/0.01 | .76 | .80 |
| SLF -II -R | 2.69 | 0.14 | 14.8% | 0.00 | 0.12/0.02 | .96 | .96 |
| SLF -III -L | 1.00 | 0.10 | 10.1% | 1.26 | 0.14/-0.01 | .26 | .33 |
| SLF -III -R | 1.00 | 0.11 | 11.2% | 1.04 | 0.11/0.02 | .31 | .37 |
| UF -L | 1.00 | 0.08 | 8.1% | 1.78 | 0.16/0.03 | .18 | .25 |
| UF -R | 1.00 | 0.09 | 9.5% | 2.41 | 0.09/0.03 | .12 | .17 |
| *Abbreviations:* FA:Fractional Anisotropy; EDF: Effective degrees of freedom-L: left; -R: right; AF: arcuate fasciculus; CB: cingulum bundle; EmC: extreme capsule; ILF: inferior longitudinal fasciculus; IOFF: inferior occipital fasciculus; MdLF: middle longitudinal fasciculus; SLF-I, II, III: superior longitudinal fasciculus I, II, III; UF: uncinate fasciculus | | | | | | | |

***2d.*** *COI Health-Environment -> Association tracts*

|  | **COI Health-Environment** | | | | | | |
| --- | --- | --- | --- | --- | --- | --- | --- |
| *Association Tracts FA* | **EDF** | **R-sq. (adj)** | **Dev Exp** | **F** | **Mean Func. / Mean Slope** | **p-value** | **FDR corrected p-value** |
| AF -L | 1.04 | 0.16 | 16.4% | 0.21 | 0.16/0.03 | .65 | .68 |
| AF -R | 1.15 | 0.10 | 10.3% | 0.65 | 0.04/0.01 | .54 | .57 |
| CB -L | 3.36 | 0.14 | 14.4% | 1.82 | 0.20/-0.01 | .11 | .16 |
| CB -R | 2.03 | 0.13 | 13.4% | 1.92 | 0.11/0.00 | .17 | .22 |
| EmC -L | 1.00 | 0.14 | 14.9% | 2.85 | 0.10/-0.01 | .09 | .13 |
| EmC -R | 1.00 | 0.14 | 14.4% | 2.90 | -0.01/-0.01 | .09 | .13 |
| ILF -L | 3.76 | 0.10 | 10.8% | 1.83 | 0.19/-0.02 | .13 | .18 |
| ILF -R | 1.19 | 0.10 | 10.3% | 1.61 | 0.11/-0.01 | .15 | .20 |
| IOFF -L | 1.44 | 0.17 | 17.9% | 0.62 | 0.03/0.01 | .40 | .45 |
| IOFF -R | 1.00 | 0.17 | 17.4% | 1.04 | 0.02/0.02 | .31 | .37 |
| MdLF -L | 1.00 | 0.14 | 14.2% | 0.90 | -0.02/0.01 | .34 | .39 |
| MdLF -R | 1.37 | 0.15 | 15.3% | 0.44 | 0.01/0.03 | .49 | .53 |
| SLF -I -L | 1.00 | **0.09** | **9.8%** | **6.66** | 0.35/0.04 | **.01** | **.02** |
| SLF -I -R | 1.00 | 0.11 | 11.2% | 1.64 | 0.32/0.04 | .20 | .26 |
| SLF -II -L | 3.80 | 0.10 | 10.0% | 1.32 | 0.23/0.03 | .26 | .32 |
| SLF -II -R | 2.85 | 0.14 | 14.9% | 0.93 | 0.13/0.03 | .36 | .41 |
| SLF -III -L | 1.21 | 0.10 | 10.1% | 0.68 | 0.13/0.00 | .35 | .40 |
| SLF -III -R | 1.00 | 0.10 | 10.8% | 0.09 | 0.12/0.01 | .76 | .78 |
| UF -L | 1.00 | 0.08 | 8.6% | 2.09 | 0.15/0.03 | .15 | .20 |
| UF -R | 1.08 | 0.09 | 9.7% | 0.71 | 0.11/0.02 | .45 | .49 |
| *Abbreviations:* FA:Fractional Anisotropy; EDF; Effective degrees of freedom: -L: left; -R: right; AF: arcuate fasciculus; CB: cingulum bundle; EmC: extreme capsule; ILF: inferior longitudinal fasciculus; IOFF: inferior occipital fasciculus; MdLF: middle longitudinal fasciculus; SLF-I, II, III: superior longitudinal fasciculus I, II, III; UF: uncinate fasciculus | | | | | | | |

# **Supplementary Table 3**. COI domain results associated with NIH Toolbox^8^ cognition scores (path c) and mediation analysis (path c’).

***3a.*** *Results for COI Education*

|  | **COI Education -> Cognition**  **Path c** | | | | | | | **Mediation Analysis**  **Path c’** | |
| --- | --- | --- | --- | --- | --- | --- | --- | --- | --- |
|  | **EDF** | **R-sq. (adj)** | **Dev. Exp.** | **F** | **Mean Func. / Mean Slope** | **p-value** | **FDR corrected p-value** | **Model 1**  **(M: SLF – I Left)** | |
|  |  |  |  |  |  |  |  | **Indirect Effect**  **(95% CI)** | **Mediation proportion** |
| **Total Cognition Composite** | 2.58 | 0.32 | 32.8% | 4.98 | 0.56/0.06 | .002 | **.003** | 0.91 (0.86 – 0.95) | 18.9% |
| **Fluid Cognition Composite** | 1.00 | 0.20 | 21.0% | 6.69 | 0.41/0.04 | .01 | **.02** | 0.68 (0.65 – 0.72) | 10.2% |
| **Crystallized Cognition Composite** | 1.00 | 0.31 | 31.8% | 15.26 | 0.49/0.05 | < .001 | **< .001** | 0.94 (0.90 – 0.99) | 6.2% |
| **Picture Vocabulary** | 5.22 | 0.29 | 29.9% | 5.55 | 0.50/0.11 | < .001 | **< .001** | 0.94 (0.90 – 0.98) | 16.9% |
| **Flanker Inhibitory Control and Attention** | 5.29 | 0.09 | 9.9% | 2.28 | 0.22/0.05 | .03 | **.04** | 0.45 (0.43 –0.48) | 19.8% |
| **List Sorting Working Memory** | 2.49 | 0.13 | 13.9% | 1.56 | 0.49/0.02 | .18 | .21 | - | - |
| **Dimensional Change Card Sort** | 4.40 | 0.12 | 12.7% | 1.31 | 0.23/0.04 | .26 | .27 | - | - |
| **Pattern Comparison Processing Speed** | 1.00 | 0.09 | 10.0% | 8.19 | 0.29/0.04 | .004 | **.007** | 0.32 (0.29 – 0.35) | 3.9% |
| **Picture Sequence Memory** | 1.00 | 0.08 | 8.7% | 0.71 | 0.15/0.01 | .40 | .41 | - | - |
| **Oral Reading Recognition** | 1.26 | 0.20 | 21.0% | 2.95 | 0.35/0.03 | .05 | .06 | - | - |
| *Abbreviations: COI, Child Opportunity Index; EDF, Effective degrees of freedom; Dex. Exp, Deviance explained; adj, adjusted FDR, false discovery rate; M: mediator; CI, 95 % Confidence Interval.* | | | | | | | | | |

***3b.*** *Results for COI Social-Economic*

|  | **COI Social-Economic -> Cognition**  **Path c** | | | | | | | **Mediation Analysis**  **Path c’** | | | |
| --- | --- | --- | --- | --- | --- | --- | --- | --- | --- | --- | --- |
|  | **EDF** | **R-sq. (adj)** | **Dev. Exp.** | **F** | **Mean Func. / Mean Slope** | **p-value** | **FDR corrected p-value** | **(M: SLF – I Left)** | | **(M: SLF – I Right)** | |
|  |  |  |  |  |  |  |  | **Indirect Effect**  **(95% CI)** | **Mediation proportion** | **Indirect Effect**  **(95% CI)** | **Mediation proportion** |
| **Total Cognition Composite** | 4.90 | 0.32 | 33.0% | 4.72 | 0.55/0.10 | < .001 | **< .001** | 0.91 (0.87 – 0.96) | 18.2% | 0.70 (0.66 – 0.74) | 13.9% |
| **Fluid Cognition Composite** | 1.00 | 0.31 | 21.1% | 12.83 | 0.39/0.06 | < .001 | **< .001** | 0.69 (0.65 – 0.72) | 5.4% | 0.52 (0.49 – 0.55) | 4.1% |
| **Crystallized Cognition Composite** | 2.74 | 0.32 | 32.0% | 10.89 | 0.49/0.10 | < .001 | **< .001** | 0.94 (0.90 – 0.99) | 8.7% | 0.74 (0.70 – 0.772) | 6.8% |
| **Picture Vocabulary** | 2.83 | 0.30 | 30.0% | 12.28 | 0.49/0.11 | < .001 | **< .001** | 0.94 (0.90 – 0.98) | 7.7% | 0.74 (0.70 – 0.773) | 6.0% |
| **Flanker Inhibitory Control and Attention** | 4.45 | 0.09 | 9.9% | 2.35 | 0.20/0.07 | .02 | **.03** | 0.45 (0.43 – 0.48) | 19.4% | 0.37 (0.34 – 0.39) | 15.7% |
| **List Sorting Working Memory** | 1.70 | 0.13 | 13.9% | 4.37 | 0.47/0.05 | .009 | **.01** | 0.68 (0.65 – 0.71) | 15.5% | 0.56 (0.53 – 0.58) | 12.8% |
| **Dimensional Change Card Sort** | 1.00 | 0.12 | 12.6% | 3.59 | 0.21/0.03 | .06 | .07 | - | - | - | - |
| **Pattern Comparison Processing Speed** | 3.42 | 0.10 | 10.3% | 3.48 | 0.22/0.03 | .007 | **.01** | 0.32 (0.29 – 0.35) | 9.2% | 0.21 (0.18 – 0.23) | 6.1% |
| **Picture Sequence Memory** | 1.07 | 0.08 | 8.7% | 1.93 | 0.14/0.02 | .18 | .19 | - | - | - | - |
| **Oral Reading Recognition** | 1.99 | 0.21 | 21.2% | 6.09 | 0.34/0.06 | .001 | **.002** | 0.69 (0.66 – 0.73) | 11.4% | 0.54 (0.50 – 0.57) | 8.8% |
| *Abbreviations: COI, Child Opportunity Index; EDF, Effective degrees of freedom; Dex. Exp, Deviance explained; adj, adjusted FDR, false discovery rate; M: mediator; CI, 95 % Confidence Interval.* | | | | | | | | | | | |

***3c.*** *Results for COI Health-Environment*

|  | **COI Health-Environment -> Cognition**  **Path c** | | | | | | | **Mediation Analysis**  **Path c’** | |
| --- | --- | --- | --- | --- | --- | --- | --- | --- | --- |
|  | **EDF** | **R-sq. (adj)** | **Dev. Exp.** | **F** | **Mean Func. / Mean Slope** | **p-value** | **FDR corrected p-value** | **Model 1**  **(M: SLF – I Left)** | |
|  |  |  |  |  |  |  |  | **Indirect Effect**  **(95% CI)** | **Mediation proportion** |
| **Total Cognition Composite** | 2.09 | 0.32 | 32.8% | 6.65 | 0.57/0.05 | < .001 | **.002** | 0.91  (0.86 – 0.96) | 13.8% |
| **Fluid Cognition Composite** | 1.24 | 0.21 | 21.1% | 7.50 | 0.39/0.05 | .006 | **.009** | 0.69  (0.65 – 0.72) | 10.7% |
| **Crystallized Cognition Composite** | 2.50 | 0.31 | 31.8% | 6.22 | 0.52/0.04 | < .001 | **< .001** | 0.94  (0.89 – 0.99) | 18.7% |
| **Picture Vocabulary** | 4.67 | 0.29 | 30.1% | 2.89 | 0.48/0.05 | .002 | **.004** | 0.93  (0.89 – 0.98) | 19.3% |
| **Flanker Inhibitory Control and Attention** | 1.00 | 0.09 | 9.6% | 0.93 | 0.22/0.02 | .34 | .34 | - | - |
| **List Sorting Working Memory** | 1.82 | 0.13 | 13.9% | 3.49 | 0.48/0.04 | .02 | **.03** | 0.67  (0.64 – 0.70) | 3.8% |
| **Dimensional Change Card Sort** | 2.00 | 0.12 | 12.7% | 2.15 | 0.21/0.04 | .08 | .10 | - | - |
| **Pattern Comparison Processing Speed** | 1.00 | 0.10 | 10.1% | 8.39 | 0.25/0.05 | .004 | **.007** | 0.32  (0.29 – 0.35) | 14.4% |
| **Picture Sequence Memory** | 1.00 | 0.08 | 8.7% | 2.47 | 0.13/0.03 | .12 | .13 | - | - |
| **Oral Reading Recognition** | 2.37 | 0.21 | 21.2% | 4.82 | 0.37/0.03 | .003 | **.008** | 0.69  (0.65 – 0.73) | 15.1% |
| *Abbreviations: COI, Child Opportunity Index; EDF, Effective degrees of freedom; Dex. Exp, Deviance explained; adj, adjusted FDR, false discovery rate; M: mediator; CI, 95 % Confidence Interval.* | | | | | | | | | |

**Supplementary Table 4.** Sensitivity analyses using linear mixed-effects models.

Results from linear mixed-effects models were conducted as sensitivity analyses to assess the stability of associations identified in the primary GAM framework. Models included random intercepts for family and site to account for sibling-relatedness and site-level clustering. Across association tracts, the Superior Longitudinal Fasciculus I (SLF-I) was the only tract showing a statistically significant association with neighborhood opportunity in these models. COI Overall was also significantly associated with multiple cognitive outcomes, with the strongest effects observed for Crystallized Cognition. Overall, effect directions and relative magnitudes were consistent with those observed in the primary analyses, supporting the tract-specific nature of the findings and indicating that the main conclusions are robust to alternative linear modeling assumptions.

***4a.*** *COI Overall -> Association tracts*

|  | **COI Overall** | | | | |
| --- | --- | --- | --- | --- | --- |
| *Association Tracts FA* | **R-sq. (adj)** | **Coefficient** | **StdError** | **p-value** | **FDR corrected p-value** |
| AF -L | 0.15 | -0.01 | 0.02 | 0.73 | 0.86 |
| AF -R | 0.09 | 0.00 | 0.02 | 0.99 | 0.99 |
| CB -L | 0.14 | -0.02 | 0.02 | 0.31 | 0.53 |
| CB -R | 0.12 | -0.01 | 0.02 | 0.61 | 0.77 |
| EmC -L | 0.14 | -0.01 | 0.02 | 0.42 | 0.63 |
| EmC -R | 0.13 | -0.03 | 0.02 | 0.04 | 0.12 |
| ILF -L | 0.10 | -0.02 | 0.02 | 0.14 | 0.32 |
| ILF -R | 0.09 | -0.01 | 0.02 | 0.70 | 0.84 |
| IOFF -L | 0.17 | 0.00 | 0.02 | 0.80 | 0.91 |
| IOFF -R | 0.16 | -0.01 | 0.02 | 0.28 | 0.51 |
| MdLF -L | 0.13 | 0.01 | 0.02 | 0.94 | 0.98 |
| MdLF -R | 0.14 | 0.01 | 0.02 | 0.78 | 0.89 |
| **SLF -I -L** | **0.09** | **0.05** | **0.02** | **0.001** | **0.01** |
| **SLF -I -R** | **0.10** | **0.04** | **0.02** | **0.02** | **0.02** |
| SLF -II -L | 0.09 | -0.01 | 0.02 | 0.83 | 0.92 |
| SLF -II -R | 0.14 | -0.01 | 0.02 | 0.49 | 0.68 |
| SLF -III -L | 0.10 | -0.02 | 0.02 | 0.35 | 0.57 |
| SLF -III -R | 0.10 | 0.01 | 0.02 | 0.67 | 0.82 |
| UF -L | 0.07 | 0.01 | 0.02 | 0.61 | 0.77 |
| UF -R | 0.09 | 0.01 | 0.02 | 0.44 | 0.65 |
| *Abbreviations:* FA; Fractional Anisotropy; -L: left; -R: right; AF: arcuate fasciculus; CB: cingulum bundle; EmC: extreme capsule; ILF: inferior longitudinal fasciculus; IOFF: inferior occipital fasciculus; MdLF: middle longitudinal fasciculus; SLF-I, II, III: superior longitudinal fasciculus I, II, III; UF: uncinate fasciculus | | | | | |

***4b.*** *COI Education -> Association tracts*

|  | **COI Education** | | | | |
| --- | --- | --- | --- | --- | --- |
| *Association Fiber Tracts FA* | **R-sq. (adj)** | **Coefficient** | **StdError** | **p-value** | **FDR corrected p-value** |
| AF -L | 0.15 | 0.01 | 0.01 | 0.59 | 0.76 |
| AF -R | 0.09 | 0.01 | 0.01 | 0.70 | 0.85 |
| CB -L | 0.14 | -0.03 | 0.01 | 0.05 | 0.15 |
| CB -R | 0.12 | -0.02 | 0.01 | 0.36 | 0.60 |
| EmC -L | 0.14 | 0.00 | 0.01 | 0.79 | 0.90 |
| EmC -R | 0.13 | -0.01 | 0.01 | 0.35 | 0.59 |
| ILF -L | 0.10 | -0.01 | 0.01 | 0.39 | 0.63 |
| ILF -R | 0.09 | 0.00 | 0.01 | 0.90 | 0.95 |
| IOFF -L | 0.17 | 0.01 | 0.01 | 0.90 | 0.95 |
| IOFF -R | 0.16 | 0.00 | 0.01 | 0.94 | 0.97 |
| MdLF -L | 0.13 | 0.00 | 0.01 | 0.73 | 0.87 |
| MdLF -R | 0.14 | 0.01 | 0.01 | 0.49 | 0.70 |
| SLF -I -L | **0.09** | **0.05** | **0.01** | **0.001** | **0.004** |
| SLF -I -R | 0.10 | 0.04 | 0.01 | 0.03 | 0.09 |
| SLF -II -L | 0.09 | 0.01 | 0.01 | 0.36 | 0.60 |
| SLF -II -R | 0.14 | 0.00 | 0.01 | 0.95 | 0.97 |
| SLF -III -L | 0.10 | 0.00 | 0.01 | 0.89 | 0.95 |
| SLF -III -R | 0.10 | 0.01 | 0.01 | 0.48 | 0.70 |
| UF -L | 0.07 | 0.02 | 0.02 | 0.47 | 0.69 |
| UF -R | 0.09 | 0.01 | 0.01 | 0.50 | 0.70 |
| *Abbreviations:* FA; Fractional Anisotropy; -L: left; -R: right; AF: arcuate fasciculus; CB: cingulum bundle; EmC: extreme capsule; ILF: inferior longitudinal fasciculus; IOFF: inferior occipital fasciculus; MdLF: middle longitudinal fasciculus; SLF-I, II, III: superior longitudinal fasciculus I, II, III; UF: uncinate fasciculus | | | | | |

***4c.*** *COI Social-Economic -> Association tracts*

|  | **COI Social-Economic** | | | | |
| --- | --- | --- | --- | --- | --- |
| *Association Fiber Tracts FA* | **R-sq. (adj)** | **Coefficient** | **StdError** | **p-value** | **FDR corrected p-value** |
| AF -L | 0.15 | -0.01 | 0.02 | 0.62 | 0.78 |
| AF -R | 0.09 | 0.00 | 0.02 | 0.86 | 0.92 |
| CB -L | 0.14 | -0.01 | 0.02 | 0.68 | 0.82 |
| CB -R | 0.12 | 0.00 | 0.02 | 0.84 | 0.91 |
| EmC -L | 0.14 | -0.01 | 0.02 | 0.57 | 0.76 |
| EmC -R | 0.13 | -0.03 | 0.02 | 0.04 | 0.12 |
| ILF -L | 0.10 | -0.02 | 0.02 | 0.19 | 0.41 |
| ILF -R | 0.09 | 0.00 | 0.02 | 0.85 | 0.91 |
| IOFF -L | 0.17 | 0.00 | 0.02 | 0.83 | 0.91 |
| IOFF -R | 0.16 | -0.02 | 0.02 | 0.20 | 0.41 |
| MdLF -L | 0.13 | 0.02 | 0.02 | 0.52 | 0.72 |
| MdLF -R | 0.14 | 0.01 | 0.02 | 0.62 | 0.78 |
| **SLF -I -L** | **0.09** | **0.05** | **0.02** | **0.01** | **0.02** |
| SLF -I -R | 0.10 | 0.03 | 0.02 | 0.06 | 0.17 |
| SLF -II -L | 0.09 | -0.01 | 0.02 | 0.64 | 0.79 |
| SLF -II -R | 0.14 | -0.01 | 0.02 | 0.42 | 0.64 |
| SLF -III -L | 0.10 | -0.02 | 0.02 | 0.29 | 0.51 |
| SLF -III -R | 0.10 | 0.00 | 0.02 | 0.67 | 0.81 |
| UF -L | 0.07 | 0.01 | 0.02 | 0.63 | 0.79 |
| UF -R | 0.09 | 0.01 | 0.02 | 0.45 | 0.67 |
| *Abbreviations:* FA; Fractional Anisotropy; -L: left; -R: right; AF: arcuate fasciculus; CB: cingulum bundle; EmC: extreme capsule; ILF: inferior longitudinal fasciculus; IOFF: inferior occipital fasciculus; MdLF: middle longitudinal fasciculus; SLF-I, II, III: superior longitudinal fasciculus I, II, III; UF: uncinate fasciculus | | | | | |

***4d.*** *COI Health-Environment -> Association tracts*

|  | **COI Health-Environment** | | | | |
| --- | --- | --- | --- | --- | --- |
| *Association Fiber Tracts FA* | **R-sq. (adj)** | **Coefficient** | **StdError** | **p-value** | **FDR corrected p-value** |
| AF -L | 0.15 | -0.02 | 0.01 | 0.13 | 0.29 |
| AF -R | 0.09 | -0.01 | 0.01 | 0.33 | 0.51 |
| CB -L | 0.14 | -0.02 | 0.01 | 0.24 | 0.44 |
| CB -R | 0.12 | -0.01 | 0.01 | 0.39 | 0.57 |
| EmC -L | 0.14 | -0.03 | 0.01 | 0.02 | 0.06 |
| EmC -R | 0.13 | -0.03 | 0.01 | 0.02 | 0.07 |
| ILF -L | 0.10 | -0.03 | 0.01 | 0.02 | 0.07 |
| ILF -R | 0.09 | -0.03 | 0.01 | 0.03 | 0.08 |
| IOFF -L | 0.17 | -0.02 | 0.01 | 0.06 | 0.16 |
| IOFF -R | 0.16 | -0.02 | 0.01 | 0.07 | 0.18 |
| MdLF -L | 0.13 | -0.02 | 0.01 | 0.04 | 0.11 |
| MdLF -R | 0.14 | -0.02 | 0.01 | 0.07 | 0.18 |
| SLF -I -L | 0.09 | 0.01 | 0.01 | 0.58 | 0.73 |
| SLF -I -R | 0.10 | 0.01 | 0.01 | 0.48 | 0.66 |
| SLF -II -L | 0.09 | -0.03 | 0.01 | 0.12 | 0.27 |
| SLF -II -R | 0.14 | -0.02 | 0.01 | 0.20 | 0.40 |
| SLF -III -L | 0.10 | -0.02 | 0.01 | 0.25 | 0.44 |
| SLF -III -R | 0.10 | -0.01 | 0.01 | 0.78 | 0.88 |
| UF -L | 0.07 | -0.01 | 0.01 | 0.56 | 0.72 |
| UF -R | 0.09 | -0.01 | 0.01 | 0.43 | 0.62 |
| *Abbreviations:* FA; Fractional Anisotropy; -L: left; -R: right; AF: arcuate fasciculus; CB: cingulum bundle; EmC: extreme capsule; ILF: inferior longitudinal fasciculus; IOFF: inferior occipital fasciculus; MdLF: middle longitudinal fasciculus; SLF-I, II, III: superior longitudinal fasciculus I, II, III; UF: uncinate fasciculus | | | | | |

***4e.*** Linear mixed-effect model for COI Overall association with cognition (path c) and mediation analysis (path c’).

|  | **COI Overall -> Cognition**  **Path c** | | | | | **Mediation Analysis**  **Path c’** | | | |
| --- | --- | --- | --- | --- | --- | --- | --- | --- | --- |
|  | **R-sq. (adj)** | **Coefficient** | **StdError** | **p-value** | **FDR corrected p-value** | **Mediation analysis**  **(M: SLF – I Left)** | | **Mediation analysis**  **(M: SLF – I Right)** | |
|  |  |  |  |  |  | **Indirect Effect**  **(95% CI)** | **Mediation proportion** | **Indirect Effect**  **(95% CI)** | **Mediation proportion** |
| **Total Cognition Composite** | 0.31 | 0.08 | 0.01 | < .001 | **< .001** | 0.04 (0.01-0.06) | 5.7% | 0.02 (0.01-0.04) | 3.5% |
| **Picture Vocabulary** | 0.29 | 0.09 | 0.02 | < .001 | **< .001** | 0.03 (0.01-0.04) | 3.8% | 0.02 (0.01-0.03) | 2.9% |
| **Flanker Inhibitory Control and Attention** | 0.08 | 0.03 | 0.02 | .06 | .08 | - | - | - | - |
| **List Sorting Working Memory** | 0.13 | 0.05 | 0.02 | .004 | **.007** | 0.03 (0.01-0.05) | 5.3% | 0.02 (0.01-0.03) | 3.3% |
| **Dimensional Change Card Sort** | 0.11 | 0.04 | 0.02 | .02 | **.03** | 0.03 (0.02-0.05) | 9.7% | 0.02 (0.01-0.03) | 4.7% |
| **Pattern Comparison Processing Speed** | 0.08 | 0.06 | 0.02 | .001 | **.002** | 0.04 (0.02-0.07) | 5.6% | 0.03 (0.01-0.075 | 3.3% |
| **Picture Sequence Memory** | 0.08 | 0.02 | 0.02 | .16 | .18 | - | - | - | - |
| **Oral Reading Recognition** | 0.20 | 0.06 | 0.02 | < .001 | **< .01** | 0.03 (0.01-0.04) | 5.7% | 0.02 (0.01-0.03) | 5.8% |
| **Fluid Cognition Composite** | 0.18 | 0.06 | 0.02 | < .001 | **< .001** | 0.04 (0.02-0.06) | 6.3% | 0.02 (0.01-0.03) | 3.2% |
| **Crystallized Cognition Composite** | 0.31 | 0.08 | 0.01 | < .001 | **< .001** | 0.03 (0.02-0.04) | 5.2% | 0.02 (0.01-0.03) | 3.9% |
| *Abbreviations: COI, Child Opportunity Index*; adj, adjusted FDR, false discovery rate; M: mediator; CI, 95 % Confidence Interval. | | | | | | | | | |

***4f.*** Linear mixed-effect model results for COI Education association with cognition (path c) and mediation analysis (path c’).

|  | **COI Education -> Cognition**  **Path c** | | | | | **Mediation Analysis**  **Path c’** | |
| --- | --- | --- | --- | --- | --- | --- | --- |
|  | **R-sq. (adj)** | **Coefficient** | **StdError** | **p-value** | **FDR corrected p-value** | **(M: SLF – I Left)** | |
|  |  |  |  |  |  | **Indirect Effect**  **(95% CI)** | **Mediation proportion** |
| **Total Cognition Composite** | 0.30 | 0.05 | 0.01 | < .001 | **< .001** | 0.03 (0.01-0.04) | 6.8% |
| **Picture Vocabulary** | 0.28 | 0.05 | 0.01 | < .001 | **< .001** | 0.02 (0.01-0.03) | 4.1% |
| **Flanker Inhibitory Control and Attention** | 0.08 | 0.02 | 0.02 | 0.20 | 0.26 | - | - |
| **List Sorting Working Memory** | 0.12 | 0.02 | 0.02 | 0.17 | 0.24 | - | - |
| **Dimensional Change Card Sort** | 0.11 | 0.02 | 0.02 | 0.13 | 0.19 | - | - |
| **Pattern Comparison Processing Speed** | 0.08 | 0.04 | 0.02 | .005 | **.01** | 0.04 (0.02-0.06) | 6.1% |
| **Picture Sequence Memory** | 0.08 | 0.01 | 0.02 | 0.56 | 0.63 | - | - |
| **Oral Reading Recognition** | 0.20 | 0.04 | 0.01 | .02 | **.03** | 0.02 (0.01-0.03) | 9.1% |
| **Fluid Cognition Composite** | 0.18 | 0.04 | 0.01 | .008 | **.03** | 0.03 (0.01-0.05) | 8.4% |
| **Crystallized Cognition Composite** | 0.31 | 0.06 | 0.01 | < .001 | **< .001** | 0.02 (0.01-0.03) | 5.5% |
| *Abbreviations: COI, Child Opportunity Index*; adj, adjusted FDR, false discovery rate; M: mediator; CI, 95 % Confidence Interval. | | | | | | | |

***4g.*** Linear mixed-effect model results for COI Social-Economic association with cognition (path c) and mediation analysis (path c’).

|  | **COI Social-Economic -> Cognition**  **Path c** | | | | | **Mediation Analysis**  **Path c’** | |
| --- | --- | --- | --- | --- | --- | --- | --- |
|  | **R-sq. (adj)** | **Coefficient** | **StdError** | **p-value** | **FDR corrected p-value** | **(M: SLF – I Left)** | |
|  |  |  |  |  |  | **Indirect Effect**  **(95% CI)** | **Mediation proportion** |
| **Total Cognition Composite** | 0.30 | 0.05 | 0.01 | < .001 | **< .001** | 0.04 (0.02-0.05) | 6.0% |
| **Picture Vocabulary** | 0.29 | 0.05 | 0.01 | < .001 | **< .001** | 0.02 (0.01-0.04) | 3.8% |
| **Flanker Inhibitory Control and Attention** | 0.08 | 0.02 | 0.02 | 0.05 | 0.08 | - | - |
| **List Sorting Working Memory** | 0.13 | 0.02 | 0.02 | 0.17 | 0.24 | - | - |
| **Dimensional Change Card Sort** | 0.11 | 0.02 | 0.02 | 0.03 | 0.05 | - | - |
| **Pattern Comparison Processing Speed** | 0.08 | 0.04 | 0.02 | 0.001 | **0.003** | 0.04 (0.02-0.05) | 4.1% |
| **Picture Sequence Memory** | 0.08 | 0.01 | 0.02 | 0.09 | 0.14 | - | - |
| **Oral Reading Recognition** | 0.20 | 0.04 | 0.01 | 0.001 | **0.002** | 0.03 (0.01-0.04) | 7.5% |
| **Fluid Cognition Composite** | 0.18 | 0.04 | 0.01 | < .001 | **0.001** | 0.02 (0.02-0.06) | 6.4% |
| **Crystallized Cognition Composite** | 0.31 | 0.06 | 0.01 | < .001 | **< .001** | 0.03 (0.02-0.06) | 5.2% |
| *Abbreviations: COI, Child Opportunity Index*; adj, adjusted FDR, false discovery rate; M: mediator; CI, 95 % Confidence Interval. | | | | | | | |

***4h.*** Linear model results for COI Health-Environment association with cognition (path c)

|  | **COI Health-Environment -> Cognition**  **Path c** | | | | |
| --- | --- | --- | --- | --- | --- |
|  | **R-sq. (adj)** | **Coefficient** | **StdError** | **p-value** | **FDR corrected p-value** |
|  |  |  |  |  |  |
| **Total Cognition Composite** | 0.30 | 0.06 | 0.02 | < .001 | **.002** |
| **Picture Vocabulary** | 0.28 | 0.04 | 0.02 | 0.01 | **0.02** |
| **Flanker Inhibitory Control and Attention** | 0.08 | 0.02 | 0.02 | 0.21 | 0.28 |
| **List Sorting Working Memory** | 0.12 | 0.03 | 0.02 | 0.10 | 0.15 |
| **Dimensional Change Card Sort** | 0.11 | 0.03 | 0.02 | 0.02 | **0.04** |
| **Pattern Comparison Processing Speed** | 0.08 | 0.05 | 0.02 | 0.001 | **0.001** |
| **Picture Sequence Memory** | 0.08 | 0.03 | 0.02 | 0.22 | 0.29 |
| **Oral Reading Recognition** | 0.20 | 0.03 | 0.02 | 0.17 | 0.23 |
| **Fluid Cognition Composite** | 0.18 | 0.06 | 0.02 | 0.03 | **0.04** |
| **Crystallized Cognition Composite** | 0.31 | 0.08 | 0.01 | 0.001 | **0.002** |
| *Abbreviations: COI, Child Opportunity Index*; adj, adjusted FDR, false discovery rate | | | | | |

# **Supplementary Fig. 1.** The figure displays the number of subjects excluded due to missing values.

**Supplementary Fig. 2.** Correlation Matrix and Variance Inflation Factors (VIF) for Included Variables to Assess Multicollinearity

**Supplementary Fig. 3. Diagnostic plots assessing the adequacy of the generalized additive model (GAM).**

Model diagnostics were examined to evaluate distributional assumptions, residual behavior, and overall model fit. **Top left:** Histogram of residuals showing an approximately symmetric distribution centered around zero, consistent with model assumptions. **Top right:** Quantile–quantile (Q–Q) plot of deviance residuals indicating close agreement with theoretical normal quantiles, with only minor deviations in the tails. **Bottom left:** Residuals plotted against the linear predictor demonstrate no systematic pattern, supporting the absence of major model misspecification or unmodeled non-linear structure. **Bottom right:** Observed responses plotted against fitted values show a monotonic relationship with no evidence of heteroscedasticity or influential outliers. Collectively, these diagnostics support the adequacy of the GAM fit and indicate that the estimated associations are not driven by violations of model assumptions or excessive model flexibility.

**Supplementary Fig. 4.** These figures display the partial effect of COI Overall on both cognitive function tests and SLF-I tracts within GAMs. The smooth line indicates the direction of the association, with a 95% confidence interval.

a. GAMs partial effect of smooth line graphs for SLF-I with COI Overall

b. GAMs partial effect of smooth line graphs for cognition

**Supplementary Fig. 5.** Associations between 29 COI indicators and NIH Toolbox Cognitive scores

**Supplementary Fig. 6.** The scatter plot shows the Child Opportunity Index 2.0 (COI) Overall score distribution across the NIH Toolbox Total Cognition Composite.

COI Overall score

Total Cognition Composite

**Supplementary References**

1.	[Fan, C. C. *et al.* Adolescent Brain Cognitive Development (ABCD) study Linked External Data (LED): Protocol and practices for geocoding and assignment of environmental data. *Dev. Cogn. Neurosci.* **52**, 101030 (2021).](http://paperpile.com/b/6gKfd1/65Dv)

2.	[Barch, D. M. *et al.* Demographic and mental health assessments in the adolescent brain and cognitive development study: Updates and age-related trajectories. *Dev. Cogn. Neurosci.* **52**, 101031 (2021).](http://paperpile.com/b/6gKfd1/puuk)

3.	[Wood, S. N. Generalized additive models: an introduction with R. (2017) doi:](http://paperpile.com/b/6gKfd1/g5W7)[10.1201/9781315370279/generalized-additive-models-simon-wood](http://dx.doi.org/10.1201/9781315370279/generalized-additive-models-simon-wood)[.](http://paperpile.com/b/6gKfd1/g5W7)

4.	[Wood, S. N. *Generalized Additive Models: An Introduction with R, Second Edition*. (Chapman & Hall/CRC, Philadelphia, PA, 2017).](http://paperpile.com/b/6gKfd1/ZfXj)

5.	[Acevedo-Garcia, D. *et al.* The child opportunity index: improving collaboration between community development and public health. *Health Aff. (Millwood)* **33**, 1948–1957 (2014).](http://paperpile.com/b/6gKfd1/s6BB)

6.	[Noelke, C. *et al.* Child Opportunity Index 2.0. Preprint at](http://paperpile.com/b/6gKfd1/6Eao) <http://new.diversitydatakids.org/sites/default/files/2020-01/ddk_coi2.0_technical_documentation_20200115_1.pdf>[.](http://paperpile.com/b/6gKfd1/6Eao)

7.	[Cetin-Karayumak, S. *et al.* Harmonized diffusion MRI data and white matter measures from the Adolescent Brain Cognitive Development Study. *Sci. Data* **11**, 249 (2024).](http://paperpile.com/b/6gKfd1/Qd8y)

8.	[Weintraub, S. *et al.* Cognition assessment using the NIH Toolbox. *Neurology* **80**, S54–64 (2013).](http://paperpile.com/b/6gKfd1/prMM)
